# Virulence, drug sensitivity and transmission success in the rodent malaria, *Plasmodium chabaudi*

**DOI:** 10.1098/rspb.2012.1792

**Published:** 2012-09-26

**Authors:** Petra Schneider, Andrew S. Bell, Derek G. Sim, Aidan J. O'Donnell, Simon Blanford, Krijn P. Paaijmans, Andrew F. Read, Sarah E. Reece

**Affiliations:** 1 Centre for Immunity, Infection and Evolution, School of Biological Sciences, University of Edinburgh, Edinburgh EH9 3JT, UK; 2 Institute of Evolution, School of Biological Sciences, University of Edinburgh, Edinburgh EH9 3JT, UK; 3 Institute of Immunology and Infection Research, School of Biological Sciences, University of Edinburgh, Edinburgh EH9 3JT, UK; 4 Department of Biology, Center for Infectious Disease Dynamics, Pennsylvania State University, University Park, Pennsylvania 16827, USA; 5 Department of Entomology, Center for Infectious Disease Dynamics, Pennsylvania State University, University Park, Pennsylvania 16827, USA; 6 Fogarty International Center, National Institutes of Health, Bethesda, MD 20892, USA

**Keywords:** competition, drug resistance, fitness, genetically diverse infection, transmission, virulence evolution

## Abstract

Here, we test the hypothesis that virulent malaria parasites are less susceptible to drug treatment than less virulent parasites. If true, drug treatment might promote the evolution of more virulent parasites (defined here as those doing more harm to hosts). Drug-resistance mechanisms that protect parasites through interactions with drug molecules at the sub-cellular level are well known. However, parasite phenotypes associated with virulence might also help parasites survive in the presence of drugs. For example, rapidly replicating parasites might be better able to recover in the host if drug treatment fails to eliminate parasites. We quantified the effects of drug treatment on the in-host survival and between-host transmission of rodent malaria (*Plasmodium chabaudi*) parasites which differed in virulence and had never been previously exposed to drugs. In all our treatment regimens and in single- and mixed-genotype infections, virulent parasites were less sensitive to pyrimethamine and artemisinin, the two antimalarial drugs we tested. Virulent parasites also achieved disproportionately greater transmission when exposed to pyrimethamine. Overall, our data suggest that drug treatment can select for more virulent parasites. Drugs targeting transmission stages (such as artemisinin) may minimize the evolutionary advantage of virulence in drug-treated infections.

## 1. Introduction

Radical alterations in parasite ecology such as that imposed by drug treatment can exert selective effects on the life-history traits and behaviours of parasites [1–5]. For example, traits that govern the growth and reproductive patterns of parasites can influence their survival and transmission in drug-treated infections [6–18]. These life-history traits need not involve direct intracellular interactions with drug molecules, yet can reduce sensitivity to treatment. They are therefore drug-resistance traits in the broadest definition. We call life-history phenotypes which confer reduced susceptibility ‘non-classical resistance’ to make a clear distinction from the traditionally studied mechanisms of drug resistance (classical resistance). Non-classical resistance traits are diverse. They include bacterial biofilms that act as drug barriers [16,17], the formation of latent stages, such as bacterial persister cells and quiescent stages in malaria parasites that survive drugs through reduced metabolic activity during exposure [9–11,18], and faster replication rates in malaria parasites and worms that may provide protection through safety in numbers or by minimizing the period when a vulnerable life-cycle stage is exposed to drugs [12,15]. Importantly, these life-history traits can also underpin both virulence and transmission [12,19–21]. We define virulence as the harm caused by parasites to their hosts. If virulence-related traits reduce drug sensitivity, drug treatment which does not clear all parasites might confer a selective advantage on more virulent parasites. This could lead to the spread of more virulent parasites in a population. This evolution would have consequences for public and animal health: not only would drug efficacy be eroded, hosts would be at greater risk of acquiring parasites that caused more severe disease.

In an earlier study with the rodent malaria *Plasmodium chabaudi*, we found that more virulent parasites had a survival advantage in infections treated with the antimalarial drug pyrimethamine [12]. In those studies, we tested virulent and avirulent genotypes from the same genetic background which had never been under drug selection. Specifically, we treated single-genotype infections with pyrimethamine that kills the asexually replicating stages (the stages responsible for disease symptoms). Here, we examine whether the survival advantage of the virulent parasites also occurs in a wider range of conditions and whether it results in increased transmission. Both in-host survival and transmission to vectors are key components of fitness for malaria parasites and so need to be examined to determine whether drug treatment could generate population-wide evolutionary change to virulence. We present a series of experiments asking the following questions: does virulence reduce sensitivity to different treatment regimens of artemisinin, an antimalarial drug which kills transmission stages as well as asexual stages [22] and is currently the frontline drug of choice in much of the world [23]? Does the survival advantage hold when parasites are competing in genetically diverse infections? What are the transmission consequences of virulence in pyrimethamine- and in artemisinin-treated infections? Our data reveal that virulence enhances in-host survival in single and mixed infections treated with either pyrimethamine or artemisinin, and when exposed to drugs, host-to-vector transmission is also enhanced for virulent parasites, an advantage that is minimized by treatment with artemisinin. Overall, our data are consistent with the hypothesis that drug treatment can select for the evolution of virulence.

## 2. Material and methods

### (a) Parasites, hosts and drugs

Hosts were 6–10-week-old C57Bl/6J female mice, with access to food and drinking water supplemented with 0.05 per cent para-amino benzoic acid [24] ad libitum. The original *P. chabaudi* isolates were obtained from thicket rats from the Central African Republic (CW) and the Republic of Congo (DK) [25]. Genotype *P. chabaudi chabaudi* CW_avir_ (CW_175_ lineage) was obtained after cloning of the wild isolate and four passages in mice. CW_vir_ (CW_202_ lineage) was derived from CW_avir_ by 11 serial passages in mice [26,27]. Serial passage of short-term infections between naive hosts results in increased virulence [28]. The virulence phenotypes of both CW genotypes are stable through freeze–thaw cycles, suggesting that their traits are based on epigenetic inheritance or fixed mutations. *Plasmodium chabaudi adami* genotype DK25p was used as a reference genotype, which was obtained from the original isolate after mosquito passage and cloning. DK was used as a reference genotype because: (i) all DK parasite stages can be distinguished from all CW parasite stages by quantitative PCR assays; (ii) it is sensitive to the drugs used in our experiments and its intrinsic drug sensitivity is the same in mixed-genotype infections with CW_vir_ or CW_avir_; and (iii) its virulence level and competitive ability are intermediate between CW_avir_ and CW_vir_ (see the electronic supplementary materials, data S1 and figure S1). None of the parasite genotypes used, or any of their ancestors, had been exposed to antimalarial drugs prior to these experiments. All mice were infected with 10^5^ parasites of either CW_avir_ or CW_vir_ and mice in the competition experiments (experiments 3 and 4) also received 10^5^ parasites of the reference genotype DK. Artemisinin (experiments 1–4) and pyrimethamine (experiment 4) were dissolved in dimethyl sulphoxide at the required concentration and injected intraperitoneally with a maximum total volume of 50 μl.

### (b) Experiments 1–3: in-host replication

In our first 3 experiments, we tested whether virulence provides an in-host survival advantage (density of asexually replicating stages) to parasites exposed to artemisinin treatment. We varied key components of drug treatment regimen (dose and duration): artemisinin treatment lasted for 4 days in experiment 1 and 1 day in experiment 2, and the doses used span from placebo to the maximum non-toxic dose for the duration of treatment as determined from pilot experiments. The drugs, doses and numbers of independent infections for each treatment group, in each experiment, are given in table 1. We tested whether virulence provides in-host survival advantages to parasites in single-genotype infections in experiments 1 and 2; and in experiment 3, we examined parasites in the more ecologically realistic scenario of genetically diverse infections. Because the avirulent and virulent CW genotypes cannot be distinguished by microscopy or molecular methods, we competed both CW_vir_ and CW_avir_ against a common genotype, DK, using the same treatment regimens (dose and duration of treatment) as for experiment 2. We monitored all infections throughout the acute phase (days 3–21) with daily measurements of red blood cell (RBC) loss, weight loss and daily collection of samples for parasite and gametocyte quantification by microscopy and/or molecular methods.

**Table 1.** Drugs, doses and number of independent infections for all experiments. Each asterisk (*) indicates a mouse euthanized early (days 9–12) in the experiment because of severe anaemia and excluded from analysis of in-host survival.

	no competition				competition			
	experiment 1		experiment 2		experiment 3		experiment 4	
	4 day duration		1 day duration		1 day duration		1 day duration	
dose^a^	CW_avir_	CW_vir_	CW_avir_	CW_vir_	CW_avir_	CW_vir_	CW_avir_	CW_vir_
placebo	5*	8***	5	7**	5	5*	4	4
ART 50	5	5	5	5	5	5	4	4
ART 100	5	5	5**	5	5	5	4	4
ART 150	5	5	—	—	—	—	—	—
ART 200	—	—	5	5*	—	—	—	—
PYR 1	—	—	—	—	—	—	4	4
PYR 3	—	—	—	—	—	—	4	4

**^a^**Dose: dose of artemisinin (ART) or pyrimethamine (PYR) in mg kg mouse^−1^ day^−1^, starting from day 5 after infection.

For experiments 1 and 2, asexual parasites were counted by microscopy. Microscopy counts were obtained from the number of parasitized RBC observed per 1000 RBC on Giemsa-stained thin smears, made from tail bleeds, and we used RBC density determined by flow cytometry to calculate parasite density per millilitre of blood. In experiment 3, asexual parasite densities for CW_vir_ or CW_avir_ and DK were calculated by subtracting the number of gametocytes from the total number of parasites detected by quantification of cDNA or DNA of the gametocyte-specific expressed gene PC302249.00.0 by genotype-specific PCR. As we are interested in the effects of drugs over the entire duration of the infections, we used cumulative CW_vir_ or CW_avir_ asexual parasite densities from the time of drug treatment to the end of the infection to investigate in-host survival and the effects of drug treatment in both single- and mixed-genotype infections. Cumulative densities of reference genotype DK were analysed to verify that in-host competition occurred (see the electronic supplementary material, data S1 and figure S1).

### (c) Experiment 4: between-host transmission

To examine the transmission consequences of drug treatment, we infected 40 mice with the reference genotype DK and either CW_avir_ or CW_vir_ as per experiment 3. These mice were treated with either placebo, a single dose of 50 or 100 mg kg^−1^ artemisinin, or with a single dose of 1 or 3 mg kg^−1^ pyrimethamine, a drug for which we have already demonstrated the in-host survival benefits of virulence [12] (table 1).

Cages of laboratory-reared *Anopheles stephensi* mosquitoes (*n* = 50) were starved overnight and allowed to feed on two anaesthetized mice (1 cage per mouse) per treatment group at day 7 post infection (one gametocyte development cycle after drug treatment) and again on days 11, 15 and 19 (*n* = 80 cages with 50 mosquitoes each) to estimate overall (cumulative) transmission success for parasites in each treatment group. Unfed mosquitoes were removed, and fed mosquitoes were kept for 7–8 days to allow fertilized parasites to develop into oocysts and produce sporozoites (the stages infective to new hosts). For each feed, 25 mosquitoes were dissected to determine the percentage of infected mosquitoes (*n* = 2000 mosquitoes). We quantified the number of genomes of CW_avir_ or CW_vir_ and DK for up to six infected mosquitoes per feed (*n* = 188 mosquitoes, from 40 feeds that resulted in infected mosquitoes) using the genotype-specific PCR assays used in experiment 3 to calculate the proportional representation of the CW_avir_ or CW_vir_ and DK alleles as a measure of relative fitness of the different genotypes. Two of the 80 feeds were excluded, one because the mouse was not infected and one because only five mosquitoes survived. Samples from all infected mice were collected on day 5, before drug treatment and on days 7, 11, 15 and 19 to verify that the dynamics of asexual and gametocyte densities were comparable in experiments 3 and 4 (see the electronic supplementary materials, data S1).

### (d) Genotype-specific quantification of parasites

For DNA extraction, RBC pellets of 5 μl blood samples, collected in citrate saline, were stored at −80°C. DNA extraction was performed using the ABI Prism 6100 and BloodPrep Kit (Applied Biosystems, Foster City, USA) according to the manufacturer's protocol [29]. For RNA extraction, 10 μl blood samples were added to 30 μl nucleic acid purification lysis solution (Applied Biosystems) and 15 μl PBS, gently mixed and stored at −80°C. RNA was extracted using the ABI Prism 6100, and cDNA was obtained by reverse transcriptase PCR (high-capacity cDNA archive kit, Applied Biosystems) according to the manufacturer's protocols [30]. For experiments 3 and 4, total parasites and gametocytes were counted using quantitative real-time PCR, using, respectively, DNA and cDNA and primers and probe based on the gametocyte-specific expressed gene PC302249.00.0. The assay was designed to distinguish parasites and gametocytes of reference genotype DK from CW, using primers DKcg2F: 5′-GGATGACTTTAGATATTTAATAAATGATTTAGAATTC-3′, DKcg3R: 5′-CTACTTTGAATTCATCTAAATGATTTGATTTT-3′ and probe DKcg2: 5′-FAM-CCAAGAGATAAAGAAAAGGTATT-MGB-3′. For direct comparison of quantification in samples from mixed-genotype infections using the same probe DKcg2, the qPCR assay that is able to distinguish CW from DK (described as specific for the detection of *P. chabaudi* genotype AS in Drew & Reece [29]) was re-designed to target the same region of the gene as the new DK-specific assay. The primers used are CWcg2F: 5′-GGATGACTTTAGATATTTAATAAATGATTTAGAATTT-3′ and CWcg3R: 5′-CTACTTTGAATTCATCTAAATGATTTGATTTC-3′. For these assays, primers were used at a concentration of 900 nM and probes at 125 nM. The performance of the qPCR was validated using microscopy-derived parasites counts. Dilution series of positive control samples, initiated from mice infected with *P. chabaudi* of genotype CW or DK, were tested in concentrations ranging from 0.022 to 1.0 × 10^4^ gametocytes µl^−1^ and 5640 to 1.5 × 10^6^ parasites µl^−1^ to enable quantification of, respectively, gametocytes and total parasites in experimental samples. The newly designed qPCR assays for CW and DK were confirmed to be genotype-specific and quantification was robust also in the presence of a range of DNA concentrations equivalent to 10^2^–10^5^ parasites of the non-target genotype. Both the CW- and DK-specific PCRs had a lower detection limit of at least 150 parasites and 10 gametocytes per microlitre of blood, and parasite and gametocyte numbers have a high correlation to threshold cycle (Ct) values from the PCR (*r*^2^ = 0.98, *p* < 0.001 for total parasites and *r*^2^ = 0.93, *p* < 0.001 for gametocytes; electronic supplementary material, figure S2).

### (e) Classical drug-resistance genotyping

Reduced sensitivity to artemisinin and its derivatives has been documented in various species of malaria parasites [31–35]. Single nucleotide polymorphisms in the ubiquitin-specific protease-1 gene (ubp1) have been repeatedly associated with lower sensitivity to artemisinins in *P. chabaudi*. Therefore, we tested CW_avir_, CW_vir_, and all samples that contained parasites at the end of our experiments for reported mutations in ubp-1 to examine whether ‘classical’ resistance arose during any experiments. Forward primer Pcpf01-0197-07: 5′-ATGCAAACTTACTTTCAAAACG-3′ and reverse primer Pcpf01-0197-04: 5′-TTGTTGCATTTCGAGCATTTG-3′ were used to amplify the region of the ubp-1 gene that contains possible mutations involved in artemisinin resistance [31,36]. PCR products were purified and sequenced in both forward and reverse direction using primers: Pcpf01-0197-08: 5′-CAAATAAAAAATATGTTTCACCAG-3′, and Pcpf01-0197-R1: 5′-CGAGCATTTGTATTTATTGTTTCC-3′ to detect presence of reported artemisinin resistance mutations in ubp-1 [31]. None of the reported artemisinin resistance mutations were detected before or after artemisinin treatment in any of the experiments.

### (f) Statistical analyses

Analyses were performed in R v. 2.11.1 (The R Foundation for Statistical Computing, Vienna, Austria). Models were minimized using step-wise deletion of the least significant term, and only significant effects are reported. To test the in-host survival of CW_vir_ compared with CW_avir_ parasites exposed to drug treatment (experiments 1–3), we used generalized linear models (GLM) with CW-specific cumulative asexual parasite counts (days 6–21 of infection) after ^10^log-transformation to meet assumptions of normality and homogeneity of variance. Full models included the main effects of virulence classification (genotype, CW_avir_ or CW_vir_), drug dose, experiment number (which controls for the effects of both longer duration of treatment in experiment 2 and of competition in experiment 3) and all two and three-way interactions. To control for baseline variation between individual mice we fitted covariates for weight and RBC density at the day of infection, and CW asexual parasite density at the start of treatment was fitted to control for possible differences in numbers of parasites present at the time of treatment.

To compare the transmission success of CW_vir_ compared with CW_avir_ parasites exposed to drug treatment (experiment 4), we undertook several analyses. First, we tested whether the virulence classification of the CW genotype in mixed infections and drug treatment determined the probability of infecting mosquitoes, and whether drug treatment disproportionally affected mosquito infection by either the CW_vir_ or CW_avir_ genotype (genotype–by-dose interaction). Because mosquito infection data are highly overdispersed, we used generalized linear mixed models using Markov Chain Monte Carlo techniques (MCMCglmm [37]; electronic supplementary material, data S2), which includes an observation-level random effect that deals with any overdispersion in the data. We fitted a binomial error structure and mouse was included as a random effect to adjust for non-independence associated with repeated sampling of each infection. Models included the main effects of virulence classification (genotype CW_avir_ or CW_vir_), drug dose and their interaction. Because it is expected that the percentage of infected mosquitoes increases with the number of gametocytes present in the bloodmeal [38], we also fitted ^10^log transformed total (CW + DK) gametocyte density at the time of mosquito feeds as a covariate. Second, we tested whether the fitness of CW (the proportional representation of CW compared with DK alleles) within infected mosquitoes was influenced by virulence classification, drug treatment and their interaction. Proportions of CW alleles were arcsine square root transformed and used as a response variable in generalized linear mixed models with the same main effects fitted as for the analysis of the proportion of mosquitoes infected and the proportion of CW gametocytes at the time of mosquito feeds was fitted as a covariate. Day nested within mouse was included as a random effect to adjust for non-independence associated with the contribution of several mosquitoes per feed on an individual mouse, and with multiple feeds that were performed on each infection at different days.

## 3. Results

We carried out four experiments to compare the effects of drug treatment on the survival and transmission of two *P. chabaudi* genotypes (from the same genetic background and not been previously exposed to drugs) that differ in virulence. Untreated infections with the virulent genotype (CW_vir_) resulted in significantly greater weight loss and anaemia compared with infections with the avirulent genotype (CW_avir_; electronic supplementary material, data S3). Drug treatments in all experiments were given from day 5 after infection, when symptoms (RBC loss and weight loss) were first observed, and consisted of either artemisinin (experiments 1–4) or pyrimethamine (experiment 4). Both drugs kill asexually replicating parasites but artemisinin also kills transmission stages (gametocytes) [22]. All treatment regimens alleviated symptoms (anaemia and weight loss) of mice infected with CW_vir_ to levels observed in untreated CW_avir_ infections (see the electronic supplementary materials, data S4). The drugs, doses and numbers of independent infections for each treatment group, in each experiment, are given in table 1.

### (a) In-host replication

In our first 3 experiments, we tested whether virulence provides an in-host survival advantage (density of asexually replicating stages) to parasites exposed to various doses and durations of artemisinin treatment, in single (experiments 1 and 2) and in the more ecologically realistic scenario of genetically diverse infections (experiment 3; table 1).

**Figure 1.**
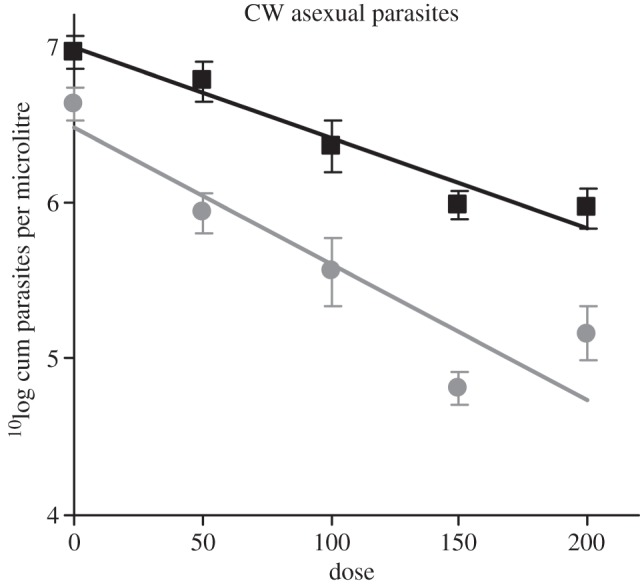
Drug sensitivity of virulent and avirulent parasite genotypes. Virulent (black squares, CW_vir_) asexual parasites are less sensitive to artemisinin treatment than avirulent (grey circles, CW_avir_) parasites in all dose and duration regimens tested. Infections were treated with placebo (0) or artemisinin at doses of 50, 100, 150 or 200 mg kg mouse^−1^. Data presented are model predictions from experiments 1–3 (mean and s.e. of cumulative asexual parasite densities from day 6–21 after infection) from GLMs controlling for weight and RBC density at the day of infection and asexual parasite density at time of treatment. Note that doses 0 (*N*_avir_ = 14, *N*_vir_ = 14), 50 (*N*_avir_ = 15, *N*_vir_ = 15) and 100 (*N*_avir_ = 13, *N*_vir_ = 15) were used in all three experiments, but doses 150 (*N*_avir_ = 5, *N*_vir_ = 5) and 200 (*N*_avir_ = 5, *N*_vir_ = 4) were only used in, respectively, experiments 1 and 2.

Infections with CW_vir_ always produced more asexual parasites than infections with CW_avir_ (*F*_1,103_ = 208.4, *p* < 0.001). Drug treatment reduced CW parasite loads (*F*_4,99_ = 83.7, *p* < 0.001) in all treatment groups, but had disproportionally greater effects on the densities of CW_avir_ compared with CW_vir_ (genotype by dose interaction: *F*_4,93_ = 5.2, *p* < 0.001; figure 1). For example, 50 mg kg^−1^ artemisinin reduced the cumulative (days 6–21) parasite densities of avirulent parasites by fivefold and that of virulent parasites only by 1.5-fold compared with placebo infections (−0.7 and −0.18 reduction on a ^10^log scale, respectively; figure 1). Therefore, CW_vir_ asexual parasites were less sensitive than CW_avir_ to artemisinin treatment in all dose and duration regimens tested. This includes: treatment of single-genotype infections for 4 consecutive days (experiment 1), for 1 day (experiment 2), and when administered for 1 day to parasites competing in mixed infections (experiment 3). This pattern was consistent across experiments despite differences in magnitude of effects generated by different treatment durations (for example, a single day treatment eliminated fewer parasites compared with giving the same dose for four consecutive days (*F*_4,89_ = 5.1, *p* = 0.001).

### (b) Between-host transmission

In experiment 4, we measured how transmission from mixed infections (as for experiment 3) is influenced by virulence and drug treatment using the same artemisinin regimens as in experiment 3 and pyrimethamine regimens according to our previous experiments [12]. We exposed infected mice to mosquitoes on day 7 post infection (one gametocyte development cycle after drug treatment) and again on days 11, 15 and 19, to estimate overall (cumulative) transmission success for parasites in each treatment group.

First, we analysed whether the probability that mosquitoes became infected—with parasites of any genotype—was influenced by the CW genotype and interactions with drug and dose. We obtained an average of 23.3 per cent infected mosquitoes per feed, ranging from 0 to 100% (table 2). However, the proportion of mosquitoes infected was not significantly influenced by the virulence of the CW genotype, drug, dose used, or by interactions between genotype and drug or dose (table 3*a*).

**Table 2.** Probability of mosquito infection. Mean and range for the percentage of infected mosquitoes throughout infections for each treatment group in experiment 4. Means were calculated across days with two feeds per treatment group done at each of four different days (*N* = 8), except in the CW_avir_ PYR 3 and ART 100 treatment groups that each had one feed excluded from analysis (*N* = 7). Treatment groups are: PYR 1: pyrimethamine 1 mg kg^–1^; PYR 3: pyrimethamine 3 mg kg^−1^; ART 50: artemisinin 50 mg kg^−1^; ART 100: artemisinin 100 mg kg^−1^.

drug dose	CW_avir_	CW_vir_
placebo	31.4 (0–92)	28.5 (0–100)
PYR 1	39.3 (0–100)	11.5 (0–92)
PYR 3	35.9 (0–92)	36.4 (0–88)
ART 50	24.1 (0–48)	12.2 (0–72)
ART 100	6.2 (0–32)	6.9 (0–36)

**Table 3.** Between-host transmission (experiment 4). (*a*) Results of MCMCglmm analysis (see the electronic supplementary materials, data S2) for the proportion of mosquitoes infected. The estimation of effects is presented (posterior mean) with 95% credible intervals (Bayesian confidence interval; CI) on a logit scale. (*b*) Results of the lme analysis of transmission success. Estimated effect sizes with standard errors (s.e.) and statistics are presented. For both sections, statistical outcomes, using CW_avir_ and no drug controls as references, with significant results indicated in bold. As doses within drug type were not significantly different, results are shown for pyrimethamine- or artemisinin-treated infections compared to no drug treatment control. Treatment groups and sample sizes (mosquito cages) are: placebo, no drugs (*N*_avir_ = 8, *N*_vir_ = 8); PYR, pyrimethamine at 1 or 3 mg kg^−1^ (*N*_avir_ = 15, *N*_vir_ = 16); ART, artemisinin at 50 or 100 mg kg^−1^ (*N*_avir_ = 15, *N*_vir_ = 16).

		posterior mean		CI	
(*a*) proportion of infected mosquitoes					
gametocyte density DK + CW		**3**.**57**		**2**.**44; 4**.**87**	
genotype (CW_vir_)		−2.10		−6.32; 2.34	
drug	PYR	0.87		−2.55; 4.75	
	ART	1.35		−2.27; 5.35	
genotype by drug interactions	CW_vir_ × PYR	−0.54		−6.21; 4.69	
	CW_vir_ × ART	−1.10		−6.91; 4.16	
		effect size	s.e.	*t*-value	*p*-value
(*b*) transmission success^a^ of CW genotypes					
proportion CW in gametocytes		**1**.**46**	**0**.**20**	**7**.**40**	**<0**.**001**
genotype (CW_vir_)		−0.27	0.19	−1.43	0.17
drug	PYR	−0.18	0.13	−1.35	0.19
	ART	−0.01	0.14	−0.03	0.97
genotype by drug interactions	CW_vir_ × PYR	**0**.**44**	**0**.**21**	**2**.**10**	**0**.**04**
	CW_vir_ × ART	0.08	0.22	0.38	0.71

^a^The proportion of CW alleles within infected mosquitoes (arcsine square root transformed).

**Figure 2.**
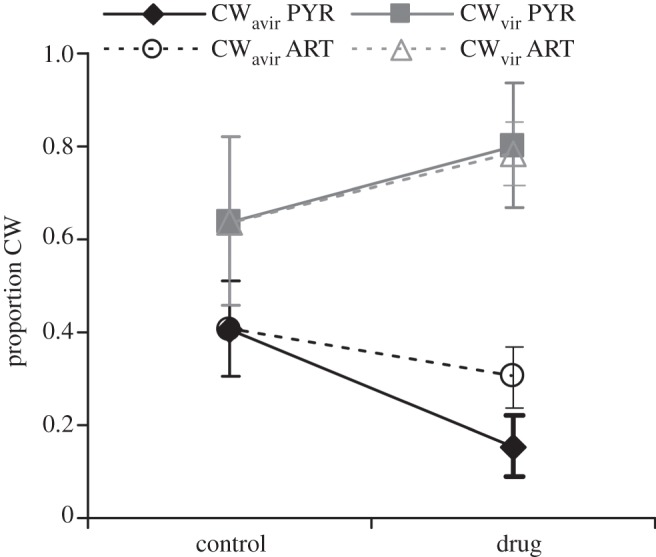
Transmission success in mixed-genotype infections. In mixed infections with a common competitor (DK), virulent (CW_vir_) parasites have a transmission (fitness) advantage over avirulent (CW_avir_) parasites, which is enhanced by treatment with pyrimethamine. Transmission success is presented as the mean ± s.e. of the proportion of CW alleles, relative to DK alleles, within infected mosquitoes. Treatment groups with sample sizes (number of feeds resulting in infected mosquitoes for avirulent and virulent CW genotypes) were: Placebo: no drugs (*N*_avir_ = 5, *N*_vir_ = 3); PYR: pyrimethamine at 1 or 3 mg kg^−1^ (*N*_avir_ = 8, *N*_vir_ = 7); ART: artemisinin at 50 or 100 mg kg^−1^ (*N*_avir_ = 8, *N*_vir_ = 8) and feeds were carried out on days 7, 11, 15 and 19 post infection.

**Figure 3.**
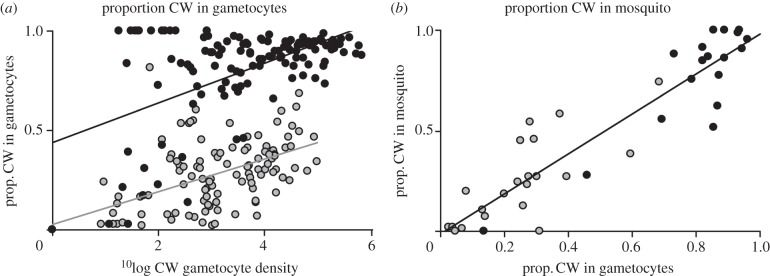
Correlation between gametocyte density, representation in the gametocyte pool and mosquito infections. The density of gametocytes (gcts) for avirulent (grey circles, CW_avir_) and virulent (black circles, CW_vir_) parasites in mixed genotype infections with common competitor, DK, correlates positively with representation in the gametocyte pool and in mosquitoes. (*a*) For a given gametocyte density, CW_vir_ (*r*^2^ = 0.31, *p* = 0.02) gains greater representation in the gametocyte pool than CW_avir_ (*r*^2^ = 0.39, *p* < 0.001). (*b*) The proportion of CW gametocytes correlates positively with the frequency of the CW allele within infected mosquitoes (*r*^2^ = 0.85, *p* < 0.001; for both genotypes). Put simply, because CW_vir_ achieves greater representation than CW_avir_ in the gametocyte pool, it gains greater representation in infected mosquitoes. Data are presented for all treatment groups ((*a*) experiment 4 and experiment 3 on days of mosquito feeds only, *N*_vir_ = 127, *N*_avir_ = 132; (*b*) experiment 4 *N*_vir_ = 18, *N*_avir_ = 21).

Second, because malaria parasites reproduce sexually in the blood meal, infected mosquitoes could harbour the CW and DK genotypes as a result of selfing, as well as progeny from recombination between the co-infecting genotypes. Therefore, we quantified how many CW and DK alleles were harboured by infected mosquitoes and used the proportion of CW relative to DK alleles as a metric to compare the transmission success of CW_avir_ and CW_vir_. The representation of CW_vir_ alleles was always greater than CW_avir_, and this difference was mediated by drug treatment (figure 2; with statistical results presented in table 3*b*). Treatment of infections with either drug did not significantly affect the transmission success of CW_vir_. In contrast, transmission of CW_avir_ was significantly reduced when infections were treated with pyrimethamine. The same pattern was observed with artemisinin, but it was not significant (table 3*b* and figure 2). We investigated whether variation in gametocyte densities could explain why CW_avir_, but not CW_vir_, suffered a reduction in transmission from drug-treated infections. First, in both treated and untreated infections, for a given gametocyte density, CW_vir_ achieved a greater representation, relative to DK, in the gametocyte pool than CW_avir_ (*F*_1,47_ = 147.77, *p* < 0.001; figure 3*a*). Second, the representation of CW in the gametocyte pool is positively and linearly correlated with allele frequency in infected mosquitoes (effect size 1.46 ± 0.20. *t* = 7.40, *p* < 0.001; table 3*b* and figure 3*b*). However, the analysis presented in table 3*b* controls for variation in the representation of CW in the gametocyte pool, which suggests that the different effects of drugs on the transmission of virulent and avirulent parasites cannot be entirely explained by variation gametocyte densities.

## 4. Discussion

Our experiments used two drugs (pyrimethamine and artemisinin), with each applied across a range of doses and regimens to avirulent and virulent parasite genotypes in single- and in mixed-genotype infections. As we found previously, the virulent parasites were less susceptible to pyrimethamine [12], and we can now report that is also true for artemisinin, an antimalarial drug with a different mode of action, in single and in genetically mixed infections (figure 1). Treatment with pyrimethamine disproportionately reduced the transmission of avirulent, compared with virulent, parasites from mixed infections (figure 2). Thus, pyrimethamine treatment generated selection which favoured the virulent genotype owing to its survival and transmission advantage. Under artemisinin treatment, the virulent parasites also experienced a disproportionate survival advantage but this did not generate a significant transmission advantage. It may be that artemisinin can also select for virulent parasites (if we had insufficient power to detect the transmission effect observed for pyrimethamine), or it may be that virulent parasites do not gain a transmission advantage with artemisinin treatment because this drug kills both asexual stages and gametocytes (pyrimethamine kills only asexual stages). However, we note that virulent parasites did not experience a transmission disadvantage, so artemisinin did not select against virulent parasites. Together, our experiments provide proof-of-principle that virulence can be associated with parasite responses to drug treatment across a range of treatment regimens. The next step is to examine whether this association holds across a broader range of drugs and genetic backgrounds—in which different traits underlie virulence—to determine whether, in general, more virulent strains are less susceptible to drug treatment. If they are, the survival advantage provided by virulence could apply to a variety of different stressors that inhibit growth (as has been shown for immunity [39] and competition [40]).

The mechanism by which virulence could reduce sensitivity to drugs in our experiments is unclear. The parasites used in our experiments had not previously been exposed to drugs and did not acquire any known classical drug resistance mutations during the experiments. This means our results are not confounded by the de novo evolution of classical drug resistance. Our analyses have controlled for the number of parasites present at the time of treatment so it is unlikely that a simple mechanism of ‘safety in numbers’ is the sole cause of the virulent parasites' advantage. However, our data do show that CW_vir_ parasites recover faster from drug treatment, which could be because CW_vir_ has a faster asexual replication rate than CW_avir_. Alternatively, dormant stages have been reported in the human malaria parasite *P. falciparum* in response to treatment with d-sorbitol, pyrimethamine, mefloquine and artemisinin-derivatives. These stages survive treatment and recover asexual replication in a dose-dependent manner up to 20 days later [9–11,18,41,42]. Our observations could be explained if virulent parasites form more dormant stages, or produce dormant stages that recover asexual replication more efficiently [43,44]. In response to our earlier report on virulence-dependent drug sensitivity to pyrimethamine [12], it was suggested that the effect may be due to overexpression or amplification of genes in the folate biosynthesis pathway, or by altered pyrimethamine transport processes [45]. These potential classical resistance mechanisms are all specifically linked to drugs that target the folate biosynthesis pathway and so are unlikely to explain the virulence-dependent drug sensitivity to artemisinin reported here.

We also found that the virulent genotype achieved greater representation (relative to a common competitor) in the pool of transmitting gametocytes (figure 3*a*) and in mosquito infections (figures 2 and 3*b*). Our analyses suggest that differences in gametocyte production between CW_avir_ and CW_vir_ are not solely responsible for this effect. Instead, the loss of infectiousness of CW_avir_ could occur if its gametocytes are more sensitive to drugs than the common competitor (DK) and/or if CW_vir's_ gametocytes are less sensitive than DK's. Alternatively, as observed in the host, CW_vir_ parasites might also replicate faster in the mosquito and produce sporozoite stages inside oocysts at a faster rate than DK.

Some field data from human malaria infections are consistent with our observations that more virulent parasites have a survival advantage in drug-treated hosts. First, it is often assumed that treatment failures are due to classical resistance mechanisms, but not all parasites surviving recommended drug treatment have classical resistance to the antimalarial drug they were exposed to [9,41,42,46,47], suggesting resistance can be genetically and phenotypically complex. The molecular mechanisms involved in artemisinin resistance are apparently not simple, and mutations related to artemisinin resistance do not fully account for delayed clearance times, which may be due to quiescent parasite stages [9–11,32,43,44]. Second, high parasite densities at the time of treatment increase the likelihood of treatment failure [48]. Third, the evolution of classical resistance has been linked to increases in cases of severe malaria and case-fatality rates [49,50]. This could be because treatment regimens reduce infection prevalence, which lowers levels of protective immunity, so that infections become rarer but result in more severe symptoms, or it could be due to the selection of virulent parasites that also have classical drug resistance. These possibilities highlight the need for virulence assays that can be used in natural systems where there are many circulating parasite genotypes, different epidemiological settings and a range of interventions being applied.

If our results apply to infectious diseases in general, they suggest the following scenario: virulent parasites are more likely to survive drug treatment. This will increase the risk of treatment failure and so, higher doses/durations of drugs may be required for radical cure. If higher drug doses are provided to control virulent parasites, then selection for virulence will be maintained. Parasite virulence may interact with the evolution of classical drug resistance in multiple, non-exclusive and potentially contradictory ways [51]: (i) because more parasites from virulent genotypes survive treatment, greater numbers provide more opportunities for classical resistance mutations to arise; (ii) as virulent parasites are able to tolerate drugs better, then there is stronger selection for classical resistance to emerge in avirulent parasites; (iii) following on, if classical resistance mutations provide a smaller benefit to virulent parasites, these mutations will spread faster in avirulent populations; and (iv) because the evolution of classical resistance is often associated with fitness costs in untreated infections [52], compensatory mechanisms may involve the subsequent evolution of traits underpinning virulence. Evaluating the occurrence of these scenarios in nature, and how this is affected by the spread of resistance and associated changes of treatment regimens will require a combination of laboratory experiments and the monitoring of molecular and phenotypic markers of virulence and resistance in natural infections. This will not be easy but is especially important for diseases, such as malaria, where virulence, classical drug resistance, and compensatory mutations involve complex and interacting phenotypes.
